# Luteinizing hormone receptor knockout mouse: What has it taught us?

**DOI:** 10.1111/andr.70000

**Published:** 2025-01-22

**Authors:** Ilpo T. Huhtaniemi

**Affiliations:** ^1^ Department of Digestion Metabolism and Reproduction, Institute of Reproductive and Developmental Biology, Hammersmith Campus, Imperial College London London UK; ^2^ Institute of Biomedicine Research Centre for Integrative Physiology and Pharmacology University of Turku, Kiinamyllynkatu Turku Finland

**Keywords:** follicle‐stimulating hormone, follicle‐stimulating hormone receptor, G protein‐coupled receptor, human chorionic gonadotropin, luteinizing hormone, luteinizing hormone receptor, luteinizing hormone/choriongonadotropin receptor, testosterone

## Abstract

Luteinizing hormone (LH), along with its agonist choriongonadotropin (hCG) in humans, is the key hormone responsible for the tropic regulation of the gonadal function. LH and hCG act through their cognate receptor, the luteinizing hormone/choriongonadotropin receptor (LHCGR; more appropriately LHR in rodents lacking CG), located in the testis in Leydig cells and in the ovary in theca, luteal, and luteinizing granulosa cells. Low levels in LHCGR are also expressed in numerous extragonadal sites. Hypogonadism is observed in humans expressing inactivating mutations in the *LHβ‐subunit* (*LHB*)and *LHCGR* genes, confirming the crucial role of LH and LHCGR in gonadal development and function. Unraveling of the *LHR* structure and the advent of gene manipulation techniques enabled the production of mouse models with inactivated *LHR* function, that is, the *LHR* knockout (LuRKO) mouse, some 20 years ago. This mouse model has thereafter been instrumental in various experimental settings, alone or combined with other genetically modified mouse models, in providing novel, and in some cases unexpected, details about the LH/LHR function. We will review here the salient findings of these studies.

## 1 INTRODUCTION

The development of gene manipulation techniques during the 1980s and 1990s (transgenics and knockouts) opened up new vistas into the exploration of gene function in vivo, still remaining the mainstay in such studies. Very early on, also the function of reproduction related genes was addressed with a variety of transgenic and knockout techniques, and major advances were made in the understanding of hormonal regulation of reproduction. Obvious targets for such studies included the numerous reproductive hormones and their receptors. At the same time, human mutations of the cognate genes were discovered, and combining the clinical and experimental observations further strengthened the acquired new information.

**FIGURE 1:**
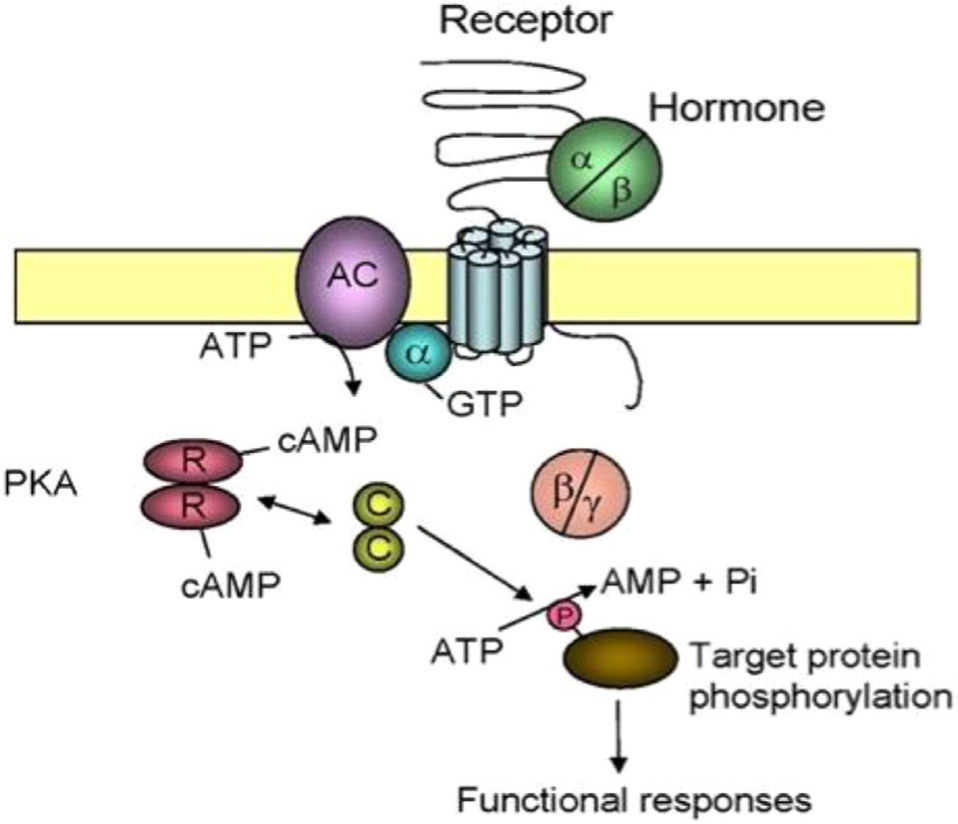
The mechanism of LH/hCG action. LH or hCG (hormone) bind to the extracellular domain of their cognate receptor, that is, LHR/LHCGR. It is a 7‐transmembrane domain G‐protein associated receptor (GPCR) with long extracellular ligand‐binding domain. Ligand binding induces association of guanidine trisphosphate (GTP) with the α‐subunit of the heterotrimeric (alpha/beta/gamma) G‐protein, thus activating the cell membrane associated adenylyl cyclase (AC) enzyme. The latter catalyzes the conversion of ATP to cyclic (c) AMP, which is the intracellular second messenger of gonadotropin action. cAMP binds to the regulatory subunit (R) of the tetrameric protein kinase A (PKA) enzyme. The liberated catalytic subunits (C) of PKA thereafter catalyze phosphorylation of target proteins (structural protein, enzymes, transcription factors), leading to alterations in their level of activation, which constitutes the most important functional response of target cells to LH/hCG stimulation. AMP, adenosine monophosphate; ATP, adenosine triphosphate; hCG, human chorionic gonadotropin; LH, luteinizing hormone; LHR, luteinizing hormone receptor; LHCGR, luteinizing hormone/choriongonadotropin receptor.

With regards to the hypothalamic–pituitary–gonadal function, human mutations were detected in genes of all the participating hormones and receptors; likewise, transgenic and knockout models were developed for nearly all of them. The *LHR* knockout was published in 2001 independently by Zhang et al.^1^(LuRKO) and Lei et al.^2^ In the former model, the last 11th exon of *LHR* was deleted, and in the latter the initial promoter and exon 1 sequences were deleted; both resulted in complete inactivation of mature *LHR* expression and luteinizing hormone (LH) signaling (Figure 1). We will cover in this review the main findings on this model in male and female mice and relate them to the findings on inactivating human *LHCGR* mutations. Also, other salient findings, where LuRKO mice were used as the experimental tool, will be reviewed.

### 1.1 Sexual differentiation and maturation of LuRKO mice

Homozygous KO mice of both sexes were born phenotypically normal.^1^ Newborn LuRKO males had similar‐sized testes, with similar intra‐abdominal location adjacent to the urinary bladder, as their wild‐type (WT) littermates. Also, the internal genitalia of the KO, heterozygous and WT males and females were anatomically indistinguishable. This is not surprising for females, because early female sex differentiation in mammals is known to occur independent of ovarian function,^3^ and ovaries start expressing gonadotropin receptors and producing functionally significant amounts of sex steroids only several days after birth.^4^

In humans, human chorionic gonadotropin (hCG) is the gonadotropin that provides the stimulus for fetal testes to produce testosterone (T), essential for male sexual differentiation.^5^ For this reason, human males with inactivating *LHCGR* mutation are not masculinized, whereas those with *LHB* inactivation—due to persistent hCG action—are normally masculinized at birth.^6^ Here, we have a clear species difference in the tropic regulation of fetal testicular function. While men devoid of luteinizing hormone/choriongonadotropin receptor (LHCGR) function are demasculinized at birth, the same is not observer in rodents. Mouse and rat fetal Leydig cells are able to respond with increased T production to a host of non‐gonadotropic hormones (e.g., ANP, PACAP, VIP), to which the adult Leydig cells are unresponsive (see, e.g., ^7^ ^,^ ^8^). Newborn genitalia are also normally masculinized in several gonadotropin deficient models, such as the *hpg* mice,^9^ the common α‐subunit KO mice,^10^ following fetal decapitation,^11^ and in *T/ebp/Nkx2.1* null mutant mice devoid of the pituitary gland.^12^ However, fetal rodent testes do express *LHR* and respond to LH stimulation,^13^ but this stimulatory link is not mandatory for their masculinization. Hence, the regulation Leydig cell function upon fetal masculinization has differential backup mechanism in humans and rodents, hCG in the former, non‐gonadotropic hormones in the latter.

### 1.2 Phenotype of adult LuRKO mice

The phenotypes of the two luteinizing hormone receptor knockout (LHRKO) mouse models, published simultaneously by us, deleting the 11th exon of *LHR*,^1^ and the group of Rao,^2^ deleting the proximal promoter and part of exon 1, were practically identical. No evidence for full‐length LHR expression was found in the KO mouse testes or ovaries at mRNA and protein level, or by radioligand binding assay.^1^ We review below the key phenotypic features of our model.

**FIGURE 2:**
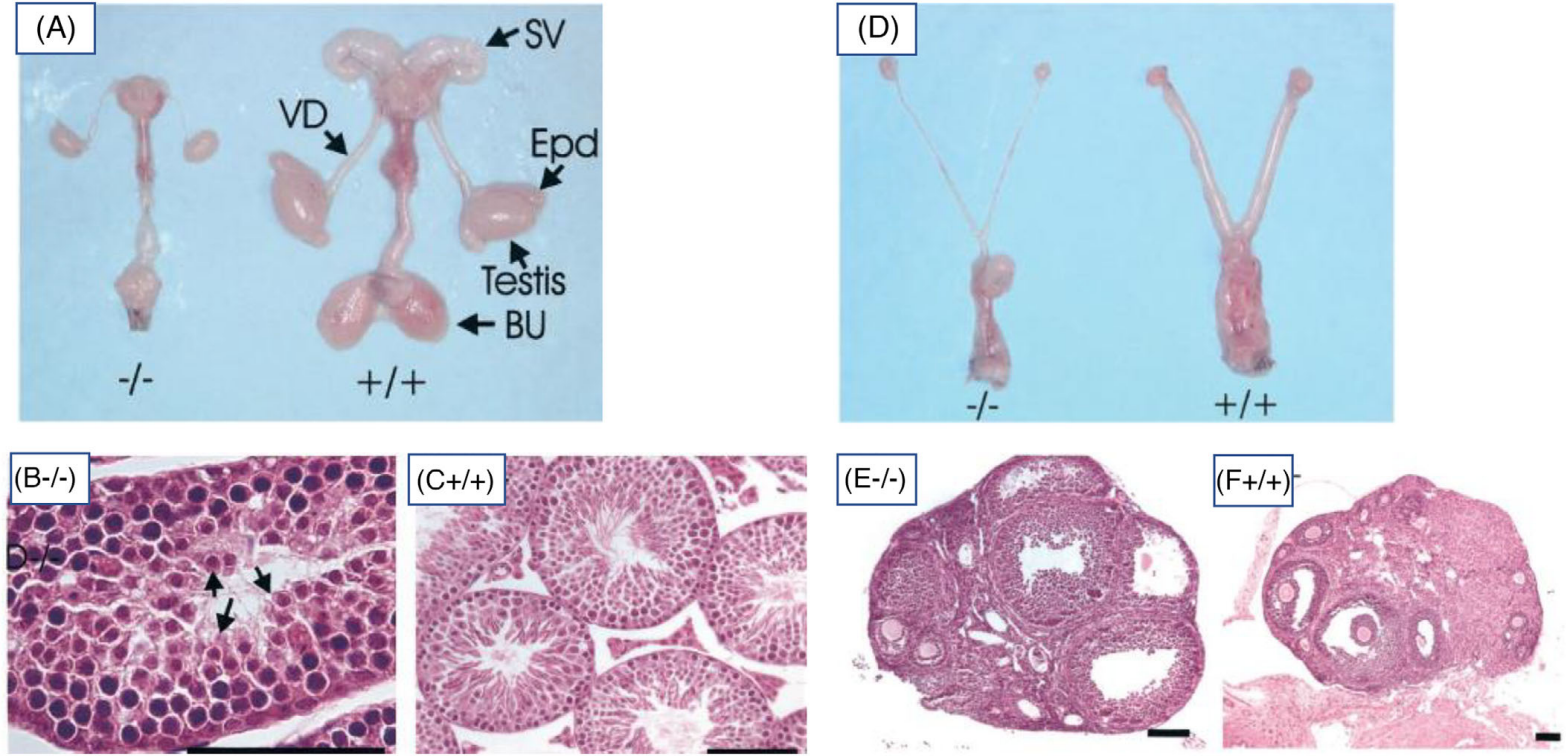
Macroscopic views of the genital structures of 7‐week‐old LuRKO (−/−) and WT (+/+) male (panel A) and female (panel B) mice. The lower sets of panels show testicular (B and C) and ovarian (E and F) histology of the same mice (−/−, LuRKO: +/+, WT). The scale bars in panels B, C, E, and F are 100 µm. From ref.^1^ WT, Wild type; LuRKO, *LHR* knockout.

The postnatal weight gain of male KO mice was suppressed, and they weighed at 3 week 30% less than the WT littermates, but such a difference was not observed between WT and KO females. The obvious reason for the weight difference was the lack of T's anabolic effect in KO males. At 30–35 days of age, the male KO mice had, in comparison to WT littermates, small penis, short anogenital distance and underdeveloped scrotum, all signs of suppressed androgen action. When studied at 7 weeks, the KO mouse testes were very small, about 20% of WT size, juxtapositioned in the abdominal cavity with the urinary bladder (Figure 2). The accessory reproductive organs (seminal vesicles, epididymides, prostate) were macroscopically invisible. Upon histological examination at 45 days of age, the testes had narrow seminiferous tubules and spermatogenesis arrested at the round spermatid stage (Figure 2). The number and size of Leydig cells were dramatically reduced.

In female LuRKO mice, vaginal opening was delayed from 30–32 days in WT to 35–38 days, apparently due to reduced estrogen levels. The ovaries of adult LuRKO females were about 50% reduced in size and the uteri were significantly thinner (Figure 2). No cyclic periodicity with recognizable estrous cycle was observed in the KO females. Ovarian histology at 7 and 12 weeks of age showed follicles up to the early antral stage, but total absence of preovulatory follicles or corpora lutea (Figure 2). In the KO mouse uteri, all cell layers were thin and there were no glandular structures. Because the ovaries of *hpg* mice have follicles up to preantral stage,^9^ it is apparent that persistent follicle‐stimulating hormone (FSH) action in the LuRKO mice can drive follicular maturation until the early antral stage. However, the lack of preovulatory follicles and corpora lutea indicates that the very last steps of follicular maturation, as well as ovulation, are strictly LH dependent. Previous studies have shown that strong FSH stimulation of hypophysectomized rodents can induce ovulation.^14^ However, this response was not found in the LuRKO mice,^15^ which we interpreted to mean that the presence of LHR, even without attached ligand, is necessary for the final follicular maturation and ovulation. The molecular nature of this apparent LHR/follicle‐stimulating hormone receptor (FSHR) co‐operation upon final stages of follicular maturation, whether through heterodimerization^16^ or though para/autocrine effects, remains to be solved.

An unexpected feature of the LuRKO ovaries was normal thickness of the thecal cell layers surrounding the follicles. Hence, although theca cells are a known target of LH action, their survival is apparently independent on LH, as has also been found in the *hpg* ovaries.^9^ However, the androgen production of theca cells, to provide substrate for granulosa cell estrogen production, is known to be LH dependent. Therefore, the apparently normal theca cells have decreased capacity of steroidogenesis, as is demonstrated by the delayed sexual maturation and hypoplastic uteri of the LuRKO females.

When assessing the potential of residual steroid hormone production of the KO testes and ovaries, we found that the mRNA of P450scc, encoding the enzyme converting cholesterol to pregnenolone, was 95% reduced in the LuRKO testes. Likewise, the mRNA of P450_17OH_, an ovarian theca cell‐specific enzyme necessary for their androgen production, was decreases by 12‐fold in the ovaries. Thus, the steroidogenic capacity of Leydig and theca cells was dramatically, though not completely, reduced. These findings suggest that LH stimulation of the ovary and testis may not activate new genes, but rather up‐regulates those with minimal expression without tropic stimulation.

As expected, the serum level of LH was dramatically, and that of FSH moderately increased in KO males and females.^1^ Serum T was also dramatically decreased, however remaining somewhat higher than in castrated males, indicating some residual T production in Leydig cells despite absent LH stimulation. As expected, ovarian estradiol and progesterone concentrations were also decreased.

In conclusion, the phenotype of the LuRKO males and females complied with what had been known about LH action and LHR function earlier, on the basis of various clinical observations and experimental studies. The LuRKO mouse provided a faithful phenocopy of LH/LHCGR inactivation in humans, with one particular exception (^17^, also see above): human males with inactivating *LHCGR* mutation are nearly completely undermasculinized at birth because of the critical role of placental hCG‐driven LHCGR stimulation of the fetal testicular T production. In rodents, in contrast, the fetal Leydig cells are in a way promiscuous, their T production responding to a host of non‐gonadotropic hormones. The hypogonadal and anovulatory phenotype of LuRKO females and women with LHCGR inactivation are very similar.

Despite the expected and somewhat lackluster phenotype of the LuRKO mouse, the model has proven very useful in further studies, often in combination with other genetically modified mice, in elucidating novel aspects, some totally unexpected, in the regulation of gonadal function. The most interesting of them will be reviewed in the following paragraphs.

### 1.3 Luteinizing hormone action, testosterone, and spermatogenesis

Spermatogenesis of the adult LuRKO males progressed until round spermatids, which complies with our knowledge that T starts driving sperm maturation beyond this stage.^18^ The residual intratesticular T concentration in LuRKO testes was 2–3 nmol/L, that is, about 2% of the WT level,^1^ ^,^ ^19^ indicating that a higher concentration is needed for the stimulation of postmeiotic sperm maturation. High‐dose T treatment, increasing intratesticular T to about 1/10 of WT concentration, was able to rescue spermatogenesis and fertility in young adult LuRKO mice.^20^

**FIGURE 3:**
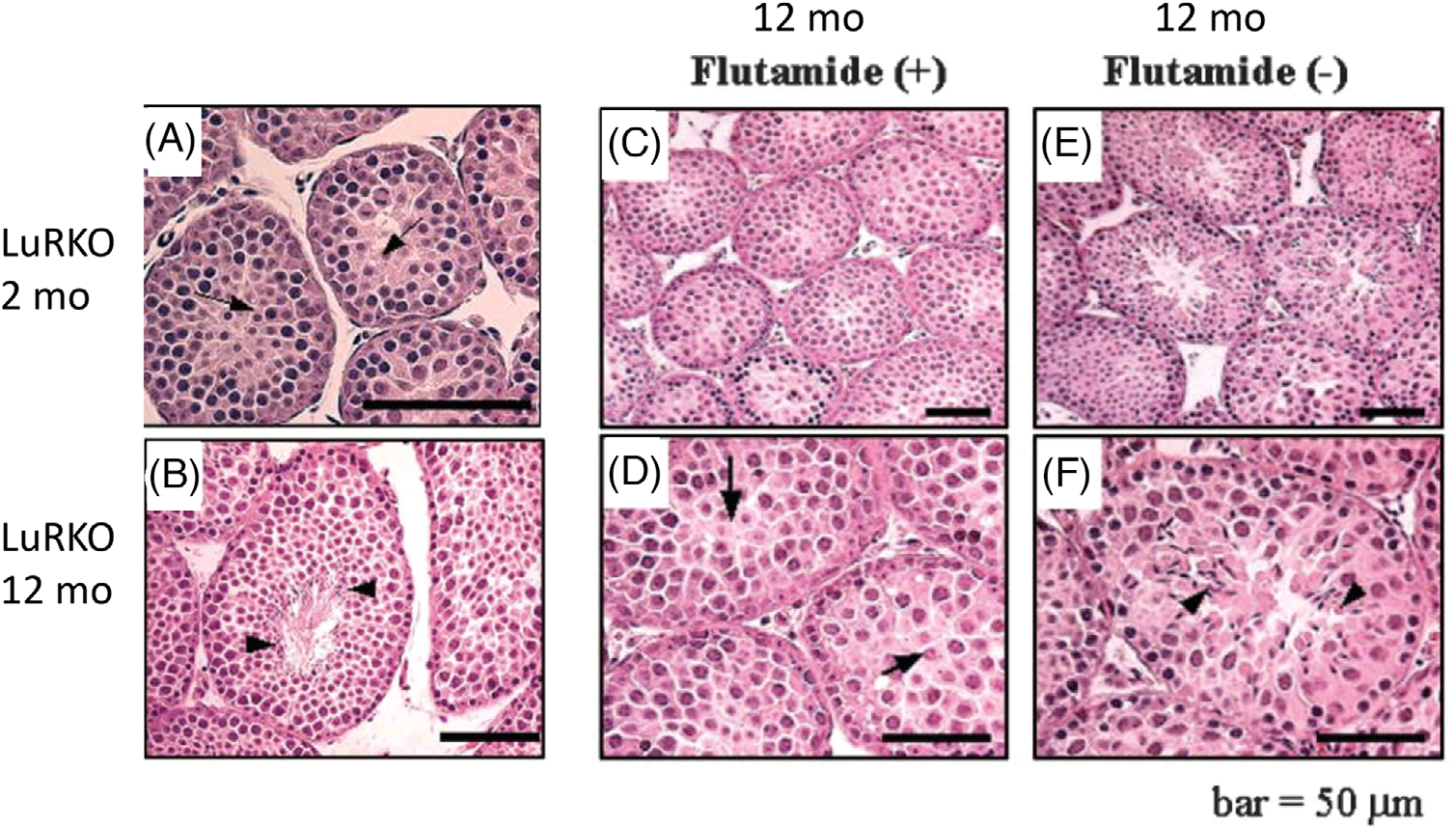
Left panels: representative light micrographs of testis sections from homozygous LuRKO testes at 2 (A) and 12 (B) months. Arrows and arrowheads indicate the most mature spermatogenic cells, round spermatids at 2 months, and elongating spermatids at 12 months. Right panels: representative light micrographs of testis sections from 12‐month‐old LuRKO mice treated with flutamide between 9–12 months (C and D). Respective control LuRKO testes (no flutamide) are in panels E and F. Flutamide treatment stopped spermatogenesis at round sprmatids, whereas in controls it proceeded to elongating spermatids. From ref.^21^ LuRKO, *LHR* knockout.

When we studied LuRKO mice at the age of 12 months, a surprising finding was made: patches of complete spermatogenesis with morphologically fully mature spermatids were present in the testes (Figure 3).^21^ Hence, besides concentration, also the duration of T exposure appears to impact sperm maturation. The low T levels present in the KO testes appeared to be responsible for the delayed sperm maturation in the 12 month‐old of mice, because it was inhibited if the mice were treated with the antiandrogen flutamide between 9–12 months of age (Figure 3). This finding provided proof for the fact that the high intratesticular T concentration, 50–100‐fold in comparison to peripheral levels, is not absolutely necessary for the maintenance of spermatogenesis, at least in the mouse, but it can also occur at a much lower concentration. The banal explanation for the high intratesticular T concentration is that it is high only because the testis is the site of T synthesis. Another explanation is that the testicular function might benefit from the immunosuppressive effect of high T concentration.

More detailed information about the dose relationship of T and spermatogenesis was obtained, when young adult LuRKO males were treated with increasing doses of T.^19^ It was found important to explore this matter in more detail, because the suppression of intratesticular T below that needed for the maintenance of spermatogenesis forms the principle of hormonal male contraception.^22^ The suppression is achieved by T treatment which, through enhanced negative feedback, suppresses gonadotropin secretion, leading to cessation of the tropic support of testicular steroidogenesis and endogenous testicular T concentration below the threshold needed for spermatogenesis.

We tested in the LuRKO mice the dose–response of spermatogenesis to graded doses of T supplementation.^19^ To this end, immature LuRKO males (22–23 days of age) were treated for 90 days with increasing doses of T from subcutaneous pellets, and the responses of their spermatogenesis and extragonadal androgen actions, including T level and gonadotropin suppression, weight gain, ano‐genital distance, balano‐preputial separation and sexual behavior, were assessed. Conspicuously, T treatment eliminated all hypogonadal signs and symptoms of the LuRKO males in a dose‐dependent manner. All the androgen‐dependent responses in the mice followed practically the same dose responses (e.g., spermatogenesis, gonadotropin suppression, and sexual function), and we could not identify a T dose that would have maintained sexual function and suppressed gonadotropins without simultaneously activating spermatogenesis.

A similar dose‐finding study on the relationship of T supplementation and suppression of spermatogenesis has never been conducted in human male contraceptive trials. The finding that all responses to T, including the extratesticular and intratesticular, appeared to have the same dose response, is in fact not unexpected because the same androgen receptor mediates androgen actions in the various sites of its expression. When extrapolated to humans, our findings may jeopardize the current approach to hormonal male contraception and call for more effective means of inhibiting intratesticular T production or action, to achieve consistent suppression of spermatogenesis. It may explain why only about 50% on Caucasian men achieve azoospermia with T treatment alone, and that supplementation of the T monotherapy with progestin is more effective, apparently through more effective suppression of gonadotropins.^22^ It remains a conundrum, why the contraceptive efficacy of T in Asian men is much better than in Europeans. One strategy to block T production more effectively would be to develop inhibitors of the HSD17B enzyme, converting androstenedione to T, but this may be difficult due to the large family of the HSD17B enzymes. Knockout of the of the most important of them contributing to testicular T production, *HSD16B1* and *HSD17B3*, did not abolish T production or spermatogenesis.^23^ ^,^ ^24^

### 1.4 Spermatogenesis without testosterone

It is textbook knowledge that LH‐stimulated high concentration of intratesticular T is a *sine qua non* for the maintenance of the sperm production.^18^ ^,^ ^25^ Human mutations and KO mice provide further proof for this, as men harboring inactivating *LHB* and *LHCGR* mutations are hypogonadal and azoospermic, with knockout mice for the same genes exhibiting similar phenotype.^17^ ^,^ ^18^ However, as described above, in particular in the mouse, the necessity of high intratesticular concentration of T has been challenged, because even T levels similar to those normally in peripheral circulation are able to maintain spermatogenesis.

The other hormone stimulating spermatogenesis is FSH. However, its role is minor in comparison to LH/T, as men with inactivating *FSHR* mutation and knockout mice for *FSHB* and *FSHR* only present with suppressed spermatogenesis, though sufficient to maintain fertility.^17^ Male mice expressing a constitutively activating *FSHR* mutation in Sertoli cells have indistinguishable phenotype from WT mice, which indicates that the maximum effect of FSH is attained at physiological levels in FSH.^26^ Two men with activating *FSHR* mutation have been described in the literature. One of them was hypophysectomized because of acromegaly, and he was thereafter found to have persistent spermatogenesis despite non‐detectable gonadotropins and low, yet higher than post‐castration, levels of T.^27^ Another activating *FSHR* mutation was identified serendipitously in an otherwise healthy man studied for testicular pain. Upon andrological examination, he exhibited normal spermatogenesis, undetectable serum FSH, and normal or elevated levels of biochemical markers for FSH action.^28^

Besides the LuRKO mouse we had produced a transgenic mouse expressing a highly constitutively activating point mutation of *FSHR* (Asp580His), targeted to Sertoli cells using the anti‐Müllerian hormone gene promoter (FSHR‐CAM).^29^ The FSHR‐CAM females presented with a strong ovarian phenotype, including hemorrhagic cysts, accelerated loss of small follicles, augmented granulosa cell proliferation, increased estradiol biosynthesis, and occasional luteinized unruptured follicles or teratomas. As stated above,^26^ no phenotype was observed in the FSHR‐CAM males, which indicated that physiological FSH stimulation attains maximum effect in males, at least when the T component of testicular stimulation is normal. These two mouse models provided us with tools to study to what extent, if any, strong FSHR activation can drive spermatogenesis in the absence of LH‐stimulated T production.

**FIGURE 4:**
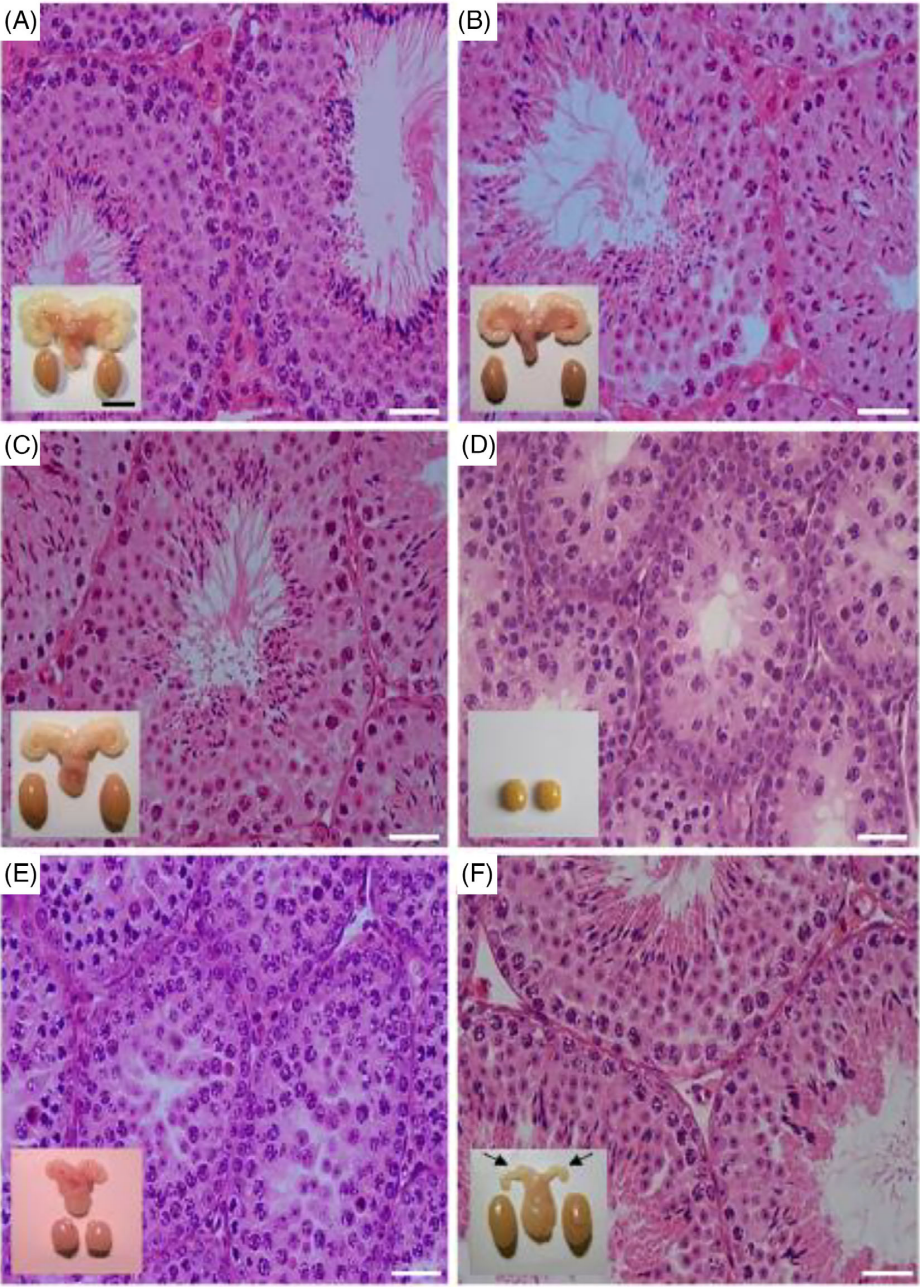
Testicular histology and macroscopic views of testes and urogenital blocks from different mouse genotypes and from flutamide‐treated animals. Representative views of (A) WT, (B) FSHR‐CAM, (C) FSHR‐CAM/LuRKO, and (D) LuRKO mice (*n* = 5–8/group). Panels (A)–(C) show normal spermatogenesis, and testis and seminal vesicle (SV) sizes. In panel (D), spermatogenesis is shown as arrested at the round spermatid (RS) stage, with small testes and rudimentary SV (not shown). (E) Treatment of WT mice (*n* = 5/group) with antiandrogen flutamide arrested spermatogenesis at the RS stage, with reduced testis and SV sizes. (F) Identical treatment of FSHR‐CAM/LuRKO mice (*n* = 5/group) had no apparent effect on spermatogenesis and testis size, but reduced SV sizes (arrows). Scale bars: 50 µm; 10 mm (insets). From Ref.^26^ WT, Wild type; LuRKO, *LHR* knockout; FSHR, follicle‐stimulating hormone receptor.

Hence, the LuRKO and FSHR‐CAM mice were crossed, and their reproductive function was assessed. Quite surprisingly, we found complete spermatogenesis and fertility in the double mutant mice (Figure 4).^26^ We initially considered the finding trivial, as we found that T levels of the double mutant mice were partially restored. There is evidence that T synthesis can be partially maintained by FSH action on Sertoli cells, which through a paracrine link indirectly brings about stimulation of Leydig cells.^30^, ^31^, ^32^

We then blocked the effect of the partially recovered T production in the double mutant mice, and WT controls, with the antiandrogen flutamide.^26^ The treatment blocked spermatogenesis in control WT mice, as expected, at the round spermatid stage. Surprisingly, spermatogenesis in the double mutant mice continued unharmed despite the blockage of androgen action (Figure 4). This finding was interpreted to mean that androgen action on spermatogenesis can be completely compensated for by strong FSHR activation. It is crucial for the stimulation of spermatogenesis in the LuRKO/FSH‐CAM mice, following the blockage of T action, that the FSHR activation is strong. Similar studies on lower level FSH stimulation of LuRKO mice^33^ or gonadotropin deficient *hpg*/androgen receptor double KO mice^34^ were not able to induce complete spermatogenesis. As an explanation for the mechanism of the unexpected similarity of FSH and T action, we found that the expression of several Sertoli cell genes, purported to be specifically androgen dependent, among them *Drd5, Rhox5* and *Eppin*,^18^ were upregulated, and unaffected by flutamide, in the double mutant mice. Detailed scrutiny of the signaling cascades activated by T and FSH shows that, indeed, there is some convergence among them, explaining their partially overlapping effects.^35^

The finding of strong spermatogenic effect of constitutively activating FSHR has human relevance. The FSH treatment of idiopathic oligozoospermia has mainly yielded equivocal results.^36^ One recent study, however, when using 3–6‐fold higher treatment doses of FSH than the usual 75 IU 2–3 times weekly, showed significant stimulation of spermatogenesis following a 5‐month treatment.^37^ The findings were clinically significant, because the sperm counts increased about 4‐fold, spontaneous pregnancy rate increased from 6% to nearly 30%, and ART pregnancy rates doubled. As in our mouse study, this clinical trial emphasized the importance of strong FSH stimulation in improving spermatogenesis. The Chinese study^37^ awaits independent verification.

The findings on the FSHR‐CAM mouse may also explain the mechanism of the two men with constitutively activating mutations of *FSHR* (see above), as well as the rare LH/T‐deficient Pasqualini syndrome (fertile eunuch).^38^ Clearly, spermatogenesis is possible without T, and the potential of strong FSH stimulation in the treatment of spermatogenic failure needs more attention. Finally, our findings provide insight into the perplexing evolutionary shift in the hormonal regulation of spermatogenesis from being FSH dominated in teleost fishes to LH/T dominance in mammals.^39^ ^,^ ^40^

### 1.5 Extragonadal luteinizing hormone receptor expression revisited

**FIGURE 5:**
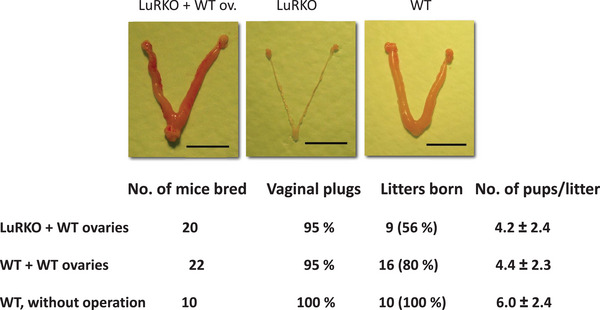
Upper panels: representative macroscopic images of the uteri and ovaries of LuRKO mice transplanted with WT ovary (left), non‐treated LuRKO (middle), and WT mice transplanted with WT ovary (right). The slight hyperemia in the LuRKO + WT ov. uterus was not a consistent finding. Lower panel: mating data of the same treatment groups. From ref.^47^ WT, Wild type; LuRKO, *LHR* knockout.

A host of studies have demonstrated low levels of LHCGR expression in humans (and LHR in rodents) in a number of reproductive and non‐reproductive organs, including uterus, oviduct, cervix, placenta, mammary gland, sperm, certain areas of brain, and many others.^41^ ^,^ ^42^ Concepts have been put forward about a wider array of LH/hCG actions beyond reproductive physiology.^43^ ^,^ ^44^ Although some effects of LH and hCG have been shown in extragonadal sites (e.g.,^45^ ^,^ ^46^) their physiological significance remains open. We addressed the function of the extragonadal LHR expression by replacing the ovaries of prepubertal LuRKO mice orthotopically with pieces of WT ovary, using similarly transplanted WT mice as controls (Figure 5).^47^ Most ovarian transplants attained the normal endocrine function in both groups of mice, as demonstrated by normal age at vaginal opening, estrous cycles, and sexual behavior. Furthermore, the transplanted LuRKO mice became repeatedly pregnant, delivered similarly sized litters with WT controls, and the pups grew normally after birth, indicating normal lactation (Figure 5). Hence, the restoration of fertility of female LuRKO mice by transplantation of WT ovarian tissue demonstrated that the extragonadal LHR expression, absent in the KO mice with ovary transplants, is physiologically redundant for female fertility. Rao et al.,^48^ using their LHR KO model, were unable to restore fertility after similar orthotopic WT ovary transplantations. Numerous differences in experimental details can contribute to the differential outcome, although, in principle, the probative value of a negative result is less than that of a positive finding. The presence of low levels of LHR expression in nongonadal sites cannot be disputed, but the importance of their function, at least for female fertility, still remains unproven. Neither did we find any signs of extragonadal phenotypes in the LuRKO mice of either sex.

There is also evidence in humans about the redundancy of the extragonadal LHCGR function. One case of successful infertility treatment through ovum donation has been reported in a woman with infertility due to compound heterozygous inactivating *LHCGR* mutation.^49^ This case demonstrates that, as in mice, extragonadal expression of LHCGR is redundant for successful pregnancy. Moreover, ovum donations bring about successful pregnancies in women with inactivating mutation in the *FSHR* gene, indicating similar redundancy of extragonadal FSHR expression.^50^

The situation in males with inactivating LHR mutation is a bit different. Human males with inactivating *LHCGR* mutation are completely unmasculinized, express immature female phenotype, female gender identity, and absence of secondary sexual maturation at puberty.^51^ Therefore, restoration of their male‐type fertility by postnatal rescue of the testicular function is not feasible. In contrast, in mice, presenting with normal male phenotype at birth but lacking postnatal masculinization in the absence of LH‐stimulated T production, the restoration of male fertility can be attempted. This can be simplest achieved by the T treatment, where a sufficient replacement dose can restore spermatogenesis, sexual function, and fertility.^19^ ^,^ ^20^ In a way, an analogous experiment with the ovarian transplantation in LuRKO female was the study, where Leydig stem cells were transplanted into LHR KO testes; the procedure partially restored T production and spermatogenesis.^52^ More recently, hypogonadism was genetically corrected in stem Leydig cells from mice carrying an inactivating LHR mutation.^53^ The T replacement and stem Leydig cell transplantation studies demonstrated that, apart from stimulating Leydig cell T production, extragonadal LHR expression is also redundant for male fertility.

### 1.6 Luteinizing hormone receptor dimerization

The old dogma is that a monomeric one ligand/one receptor complex constitutes the functional unit of G protein‐coupled receptor (GPCR) signaling, at least as it concerns class A GPCRs, such as LHR and FSHR. There is increasing functional evidence from in vitro studies that class A GPCRs could transduce their signal also as di/oligomers, thereby diversifying and/or biasing signaling functions.^54^ ^,^ ^55^ Gonadotropin receptors, because of their structure, are particularly suitable for studying this functional mode, since their lingand‐binding and signaling domains are physically separate (Figure 1). The former is in the long extracellular tail of the receptor, and the latter in the heptahelical transmembrane domain. Accordingly, co‐expressing in vitro LHR mutants, carrying mutations either in the signaling or the ligand‐binding receptor domains, was found to partially restore LHR function (see, e.g.,^56^, ^57^, ^58^). In these experiments, the mutant receptors, nonfunctional on their own, were “forced” to achieve functional complementation through dimerization, also termed transactivation through intermolecular cooperation. Because of existence of this in vitro information, we set out to find out whether the same phenomenon could be observed in vivo in LHR mice, thus emphasizing its physiological significance.

**FIGURE 6:**
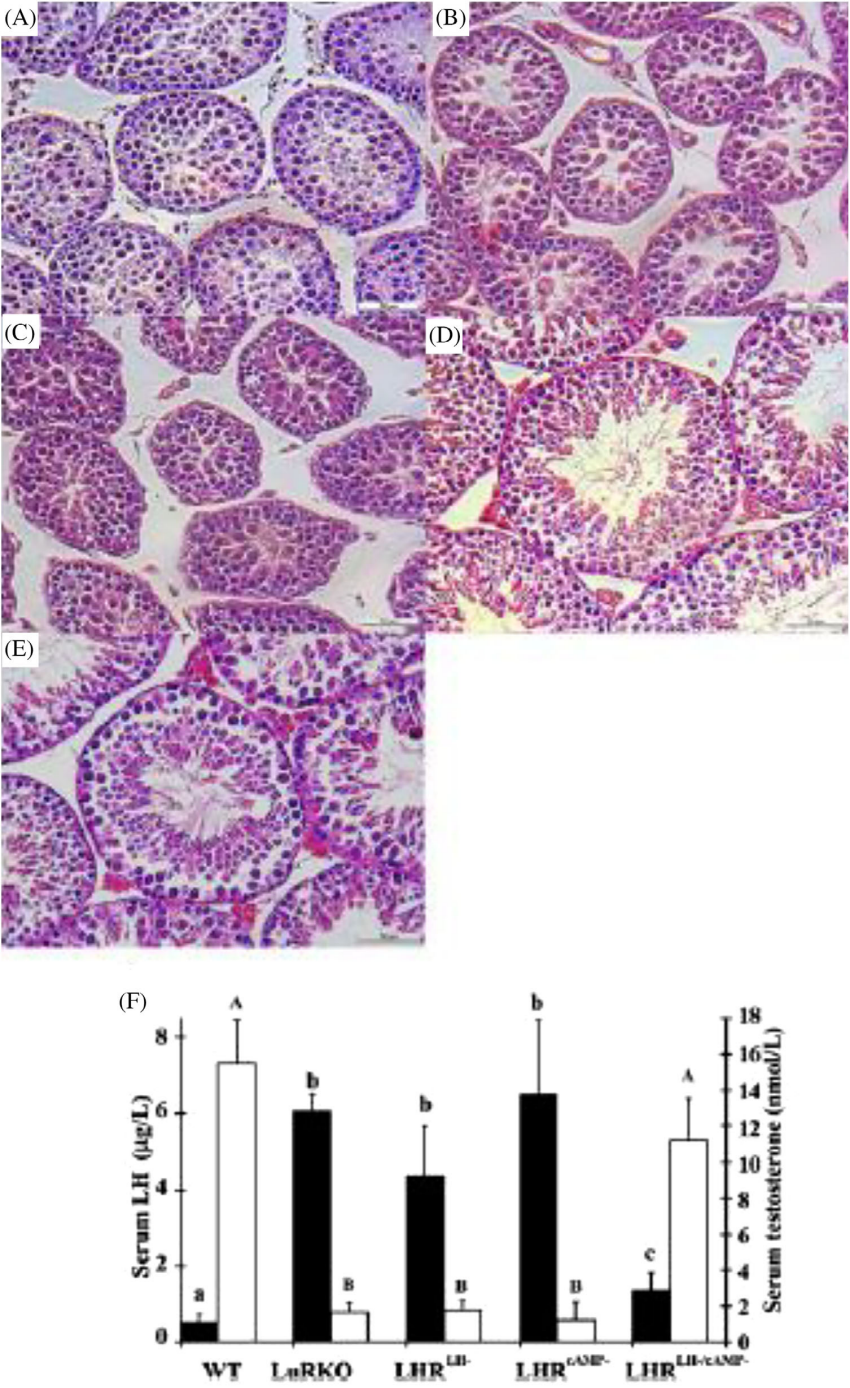
Upper panels: testicular histology of the WT (e), LuRKO (a), and LuRKO mice expressing the binding deficient (LHR^LH−^) (b) or signaling‐deficient (LHR^cAMP−^) (c) LHR mutants either alone or combined (LHR^LH‐cAMP‐)^(d). Scale bars: 50 µm. Lower panel: serum levels of LH (filled bars) and testosterone (open bars) in the same experimental groups. Each bar denotes the mean ± SD of measurements from at least four mice. Different letters above the bars indicate that these levels differ significantly (*P* at least < 0.05). From ref.^58^ WT, Wild type; LuRKO, *LHR* knockout; LHR, luteinizing hormone receptor.

**FIGURE 7:**
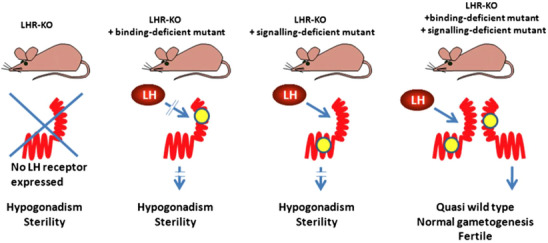
Schematic representation of the strategy to demonstrate in vivo dimerization of LHRs. The various genotypes used are presented in the top of the figure. The binding‐deficient or signaling‐deficient receptors are schematized with mutations (yellow dot) affecting the ectodomain or the transmembrane domain, respectively. From ref.^60^ LHR, Luteinizing hormone receptor.

To explore, whether functional complementation through dimerization is a physiologically meaningful mechanism of the LHR function, we took advantage of the LuRKO mice. We developed two new bacterial artificial chromosome (BAC) transgenic mouse models, one expressing a ligand‐binding deficient LHR mutant (through a point mutation in exon 1), and another one expressing a signaling‐deficient BAC‐LHR mutant (deletion of a part of exon 11).^59^ These mice, fertile as heterozygotes, were mated with likewise fertile heterozygous LuRKO mice, and after several rounds of mating, mice co‐expressing heterozygously both of the BAC transgenes in the homozygous LuRKO background were obtained. It was found that the triple‐mutant males displayed complete reversal of the hypogonadal phenotype of the LuRKO males. Testicular size and histology, T production, and fertility were indistinguishable from WT control mice (Figure 6). These results provided compelling in vivo evidence for the physiological relevance of intermolecular cooperation in LHR signaling, which is the only thinkable explanation for the findings. Besides providing an alternative molecular model for signaling, di/oligomerization could contribute to negative or positive receptor cooperativity, reconstitution of activation by mutant receptors, and ligand promiscuity. The summary of these findings is presented in Figure 7.^60^

Interestingly, when we studied the female mice, no rescue of anovulatory infertility of the LuRKO females was achieved with co‐expression of the binding‐ and signaling‐deficient LHR mutants.^61^ The regulation of the LHR expression is much more dynamic and complex in the ovary than testis, requiring induced expression in granulosa cells in the mature large antral follicles,^62^ ^,^ ^63^ the activation of multiple G protein‐dependent pathways^64^ ^,^ ^65^ and transactivation of the epidermal growth factor receptor,^66^ ^,^ ^67^ for initiating the key ovulatory pathway networks.

While only a small amount of LHR signaling (< 1% receptor occupancy) is sufficient to trigger T generation and to rescue spermatogenesis, the cyclical changes and more complex LHR actions in the ovary need stronger LHR activation, which is not attained by the relatively ineffective activation of LHR signaling upon dimerization‐associated functional cooperation of the two inactive LHR mutants. It is apparent that the need of very low level of functional receptor complexes is limited to the stimulation of Leydig cells steroidogenesis. There is evidenced for this from older studies on the “spare receptor concept,” stipulating that less than 1% of the physiological constituent of LHR need to be occupied to evoke maximum T response.^68^

The intermolecular cooperation upon LHR activation might provide answers to some questions hotly debated about GPCR di‐ and oligomerization,^69^ ^–^ ^71^ because it suggests that the receptors, when in close physical contact, can activate another or several others in their vicinity after ligand binding. It could also contribute to the “spare receptor” concept, that is, that a small proportion of liganded receptors is sufficient to evoke full biological response. Most importantly, however, intermolecular cooperation may also occur between other GPCRs, adding to the complexity of these receptor interactions and their diversity in biological systems.

### 1.7 Conclusions and future perspectives

The phenotype of LuRKO mice was quite expected, and phenocopied in mice what is known in humans about LH/hCG action. The clearest difference was the phenotype between neonatal LuRKO males and humans with inactivation mutation in *LHCGR*. While fetal sexual differentiation in the KO mice proceeded normally, due to LH‐independence of rodent fetal Leydig cells, male‐type fetal sexual differentiation in humans was completely blocked in the absence of the LHCGR function.

Transplantation of WT ovarian tissue to LuRKO females restored their fertility, T replacement restored spermatogenesis and fertility of KO males, and no extragonadal phenotypes were found in either sex. These findings cast doubt on physiological role of the low extragonadal LHR expression which is apparently real, first discovered in porcine uterus in 1986.^72^ A similar controversy surrounds the high‐profile data on extragonadal FSH actions, based largely on findings of one research group (see, e.g.,^73^).

With regards to spermatogenesis, the T treatments of LuRKO mice, and their crossing with the FSHR‐CAM mice revealed entirely novel information. It appeared that, following T replacement, the intratesticular T concentration need not be more than the physiological level in peripheral circulation, that is, a few nmol/L, to drive qualitatively and quantitatively complete spermatogenesis. This casts doubts on the importance of the normally high intratesticular T concentration as being vital for sperm production. The full spermatogenesis in LuRKO/FSHR‐CAM double mutant mice, even after blockage of androgen action with the antiandrogen flutamide, reveals a new feature of FSH action, whereby strong FSHR activation can drive spermatogenesis without T. This finding can be applied to clinical andrology, in the form of high‐dose FSH treatment of idiopathic oligozoospermia.

The demonstration of LHR dimerization and transactivation in vivo, shown previously only in vitro, to be a functional mode of receptor function, alludes to the flexibility and variability of GPCR function, that may have relevance in drug design. More research is needed to find out whether GPCR di/oligomerization could be involved in phenomena like the spare receptor concept, biased signaling and co‐operation between different types of receptors. Recent information about the cryo‐electron microscopy structures of LHR and FSHR complexed with their natural ligands and allosteric agonists has opened up new vistas into the understanding of gonadotropin signaling at the molecular level.^74^ ^,^ ^75^

A number of poorly explored issues of the LHR function still remain. Extragonadal LHR and LHCGR expression is most likely a real phenomenon, and some extragondal effects of LH and hCG have been shown both in vitro and in vivo, but the physiological and pathophysiological significance of these, often marginal, effects still remains obscure. The need of only low levels of T for the maintenance of mouse spermatogenesis may be relevant for the attempts to develop a human male contraceptive based on the blockage of gonadotropin secretion. The difficulty to attain sufficient sperm suppression may be related to the fact that sufficient suppression of intratesticular T is not attained, as it may have to be lower than expected before spermatogenesis stops.

One of the most intriguing findings of these studies was the demonstration of T independent, FSHR driven spermatogenesis. It would be interesting to repeat this experiment in crossbreeds of *Tfm* (androgen receptor inactivation) and *FSHR‐CAM* mice, to find out if the strong FSH action could also compensate for the organizational effects of T during the fetal period of male sexual differentiation.

## AUTHOR CONTRIBUTIONS

Ilpo Huhtaniemi, as the only author, is responsible for the entire content of this review.

## Data Availability

The data that support the findings of this study are available from the corresponding author upon reasonable request.
